# The protective and toxic role of neuromelanins in brain aging and Parkinson’s disease

**DOI:** 10.1186/2193-1801-4-S1-P56

**Published:** 2015-06-12

**Authors:** Luigi Zecca, Chiara Bellei, Pierluigi Mauri, Luigi Casella, Carolina Cebrián, David Sulzer, Fabio A Zucca

**Affiliations:** Institute of Biomedical Technologies, National Research Council of Italy, 20090 Segrate (Milano), Italy; Department of Chemistry, University of Pavia, Italy; Department of Neurology, Columbia University Medical Center, USA; Departments of Psychiatry, Neurology and Pharmacology, Columbia University, USA

**Keywords:** neuromelanin, aging, Parkinson’s disease

Neuromelanins (NM) are a family of compounds occurring in all brain regions of human brain. These pigments are contained in special lysosomes together with lipid bodies, protein matrix, and accumulate in aging (Zucca et al. *Neurotox Res* 2014). The synthesis of NM is a protective process because the melanic component is generated through the removal of reactive/toxic quinones that would otherwise cause neurotoxicity (Sulzer et al. *PNAS* 2000). NM serves an additional protective role through its ability to chelate and accumulate metals, including environmentally toxic metals such as mercury and lead. Other metals like Fe, Zn, Al, Cr and Mo are also accumulated by NM (Zecca et al. *PNAS* 2008). However NM can play also a toxic role in Parkinson’s disease (PD), when it is released by dying neurons of substantia nigra. Extracellular NM particles induce microglial activation with production of superoxide, nitric oxide, hydrogen peroxide, pro-inflammatory factors and causes neurodegeneration (Zhang et al. *Neurotox Res* 2011). A high content of major histocompatibility class I complex (MHC-I) was found in NM-containing organelles of the neurons in substantia nigra (SN) and locus coeruleus (LC) which degenerate in PD, while other neurons not targeted by PD has low MHC-I. The latter can bind antigens derived from foreign proteins, presenting them on neuronal membrane. Then CD8+ cytotoxic T-cells, which were observed in proximity of MHC-I presenting neurons of SN and LC in PD subjects, can target these neurons inducing neuronal death. Infiltration of T-cells occurs in SN and LC of PD subjects. The presence of MHC-I in catecholamine neurons containing NM could explain their selective vulnerability in PD, revealing a novel inflammatory T-cell mediated neurodegenerative process of PD (Cebrián et al. *Nat Commun* 2014). In conclusion NM can play a protective or toxic role depending on the molecular/cellular context.

